# Logistic regression analysis of environmental and other variables and incidences of tuberculosis in respiratory patients

**DOI:** 10.1038/s41598-020-79023-5

**Published:** 2020-12-14

**Authors:** Ashutosh K. Pathak, Mukesh Sharma, Subodh K. Katiyar, Sandeep Katiyar, Pavan K. Nagar

**Affiliations:** 1 Department of Civil Engineering and Centre for Environmental Science and Engineering, Indian Institute of Technology Kanpur, Kanpur, 208016 India; 2 Dr. S K Katiyar Chest Care Centre, Kanpur, 208002 India

**Keywords:** Environmental impact, Environmental impact, Health occupations, Risk factors

## Abstract

The objective of this study was to examine the association of 14 variables with TB in respiratory patients. The variables included: urban/rural, persons in 1200 sqft area, TB in family, crowding, smoking (family member), gender, age, education, smoking, workplace, kitchen location, cooking fuel, ventilation, and kerosene uses. Eight hundred respiratory patients were tested for sputum positive pulmonary TB; 500 had TB and 300 did not. An analysis of the unadjusted odds ratio (UOR) and adjusted OR (AOR) was undertaken using logistic regression to link the probability of TB incidences with the variables. There was an inconsistency in the significance of variables using UOR and AOR. A subset model of 4 variables (kerosene uses, ventilation, workplace, and gender) based on significant AOR was adjudged acceptable for estimating the probability of TB incidences. Uses of kerosene (AOR 2.62 (1.95, 3.54)) consistently related to incidences of TB. It was estimated that 50% reduction in kerosene uses could reduce the probability of TB by 13.29% in respiratory patients. The major recommendation was to replace kerosene uses from households with a supply of clean fuel like liquid petroleum or natural gas and rural electrification.

## Introduction

Tuberculosis (TB), caused by *Mycobacterium tuberculosis* (MTB) bacteria (also known as Tubercle bacillus), is one of the important contagious diseases and regarded as one of the major causes of global mortality and morbidity^1^. In the year 2018, a total of 1.5 million peoples worldwide died from TB and it is in the top 10 death-causing diseases^2^. The prevalence of TB is related to several variable, including low socio-economic status, immune-compromised conditions, smoking habits, the spread of multi-drug resistant TB (MDR-TB) and environmental factors^3–9^. The current research focuses on environmental and other variables to help develop policies and actions for effective control of TB.

The environmental variables such as uses of solid fuels coupled with poor indoor air quality are the common cause of compromised immunity and a large number of incidences of TB^10–14^. Worldwide, South Asia accounts for 44% of TB cases, followed by Africa (24%) and Western Pacific (18%)^2^.

Several studies^15–21^ have shown that exposure to fine particulate matter (PM_2.5_; particles of size less than or equal to 2.5 microns) from burning biomass, smoking, kerosene fuel, vehicular pollution, etc. trigger the risk of TB infection and full-blown disease. The use of kerosene, especially in the lamps for lighting purposes, have been identified to have a significant association with TB^18,22–25^. Combustion fumes also impair alveolar macrophages function, which trigger the MTB infection and damages the defence mechanism against the bacteria^16,17,26–28^.

About 41% of the global population depends on solid fuels, comprising animal dung, wood, crop residues, and coal for cooking^29^. In India, until recently, approximately 800 million individuals (70% of the total population)^30^ living in 160 million households depend on solid fuel as a primary fuel for cooking. Further, a survey from 13 states^31^ has reported that the major preferred fuel for 69% of the household was kerosene. More than 65% of rural households in the states of Uttar Pradesh and Bihar are dependent on kerosene lamps as their primary source of lighting^32^. The household air pollution is increasing due to uses of solid fuels and kerosene, making a large population vulnerable to TB infection^8,11,13,15,33,34^.

There have been recent government schemes for liquid petroleum gas connections (*Ujjwala* scheme: https://www.india.gov.in/spotlight/pradhan-mantri-ujjwala-yojana#tab=tab-1) to rural areas and *Saubghagya* scheme (https://saubhagya.gov.in/) to provide 22 million electricity connections to below poverty line households. Once data on the effectiveness of the schemes become available, the future studies could establish the improvements in air quality and reduction in TB incidences.

In addition to biomass fuel, other variables such as age, gender, work environment, smoking, kitchen ventilation, etc. have also been studied to find the association with tuberculosis^28,35–41^.

Most of the studies noted above^8,11,13,15,28,33–41^, use only a subset of variables and lack comprehensive analysis. These studies rely on the unadjusted odds ratio (UOR). The UOR depicts the odds that an outcome occurs for a particular variable, compared to the odds of the outcome occurring in the absence of that variable^42–44^. Generally, in real life, a clear relationship between a single variable and an outcome does not exist. In the case of incidences of TB, many other variables might have a role. One can adjust the UOR by controlling other variables, resulting in an adjusted odds ratio (AOR) for each variable^45^. It is important that for TB incidences, many variables coexist and the focus should be on AOR rather than UOR applied in many studies^46–48^. It is important to understand the degree of association of each coexisting variables and translate the understanding to generate a net probability of getting TB. Such analyses can also earmark the variables that should be addressed to prevent TB from progressing.

The most variables in this research relate to air pollution exposure and respiratory infection. Respiratory infection increases the risk of TB development^49^. For example, cigarette smoking impairs antimycobacterial immune responses in mice^50^.

Considering the major path of TB incidence is through a compromised immune system, this study was undertaken from the patients visiting the Chest Care Centre (CCC), Kanpur. This research's objective was to study the association of 14 independent variables (Table 1) under five categories (family, personal, occupation, kitchen and use of kerosene for cooking and lighting) with the occurrence of TB in the patients having pre-existing respiratory ailments. The city of Kanpur (longitude: 88° 22′ E and Latitude: 26° 26′ N) has reported having one of the highest numbers of TB patients (18,218)^51^ in India. Therefore, the subjects for this study were drawn from Kanpur city and nearby areas, in the state of Uttar Pradesh (UP), who visited CCC. It is clarified that all subjects had pre-existing respiratory ailments and analysis and results in the research refer to such subjects. The subjects participated in the questionnaire-based survey (described in “Methodology” section) and the data were ethically recorded for statistical analysis.

**Table 1:** UOR for Independent Variables and significance levels.

S. no.	Independent variable (abbreviations)	Groups within variable	500	300	UOR (95% CI)	Studies number (1–16)*	
						Significant	Non-significant
			Cases	Control			
** (a) Family category**							
1	Residence—Urban/Rural (RUR)	Urban	265 (53%)	162 (54%)	1	4, 14, 16	1, 7, 9, 15
		Rural	235 (47%)	138 (46%)	1.04 (0.78, 1.38)		
2	Family members in 1200 sqft of area (FMW)	≤ 5	146 (29%)	117 (39%)	1	3, 4	–
		> 5	354 (71%)	183 (61%)	1.55 (1.14, 2.09)		
3	TB in the family (TBF)	No	297 (59%)	215 (72%)	1	1, 2, 3, 4, 7, 8, 11, 14	–
		Yes	203 (41%)	85 (28%)	1.73 (1.27, 2.35)		
4	Crowding per room (CPR)	≤ 2	217 (43%)	152 (51%)	1	1, 3, 8	4, 7, 9, 10, 14, 15
		> 2	283 (57%)	148 (49%)	1.34 (1.00, 1.78)		
5	Smoking by a family member (SFM)	No	279 (56%)	172 (57%)	1	2, 14, 15	5, 7, 10
		Yes	221 (44%)	128 (43%)	1.06 (0.79, 1.42)		
** (b) Personal category**							
6	Gender (GEN)	Male	270 (54%)	193 (64%)	1	11, 12, 15	4, 5
		Female	230 (46%)	107 (36%)	1.53 (1.14, 2.06)		
7	Age (AGE)	≤ 30	199 (40%)	142 (47%)	1	3, 4, 15	7, 14
		> 30	301 (60%)	158 (53%)	1.36 (1.02, 1.81)		
8	Education (EDU)	Literate	330 (66%)	223 (74%)	1	1, 2, 14	4
		Illiterate	170 (34%)	77 (26%)	1.49 (1.08, 2.05)		
9	Smoking (SMO)	No	300 (60%)	204 (68%)	1	3, 4, 6, 7, 8, 10, 11, 14, 15	–
		Yes	200 (40%)	96 (32%)	1.42 (1.05, 1.91)		
** (c) Occupation category**							
10	Workplace (WPL)	Clean environment (CEW)	170 (33%)	133 (44%)	1	8, 14	–
		Polluted environment (PEW)	330 (66%)	167 (56%)	1.55 (1.55, 2.07)		
** (d) Kitchen category**							
11	Kitchen location (KLO)	PIH	185 (37%)	148 (49%)	1	9, 14	7
		NPIH	315 (63%)	152 (51%)	1.66 (1.24, 2.21)		
12	Cooking fuel (CFU)	LPG	169 (34%)	136 (45%)	1	6, 10, 11, 12, 13, 14, 15, 16	7, 9
		Solid fuels	331 (66%)	164 (55%)	1.62 (1.21, 2.17)		
13	Ventilation (VEN)	Yes	255 (51%)	204 (68%)	1	8, 9, 14	–
		No	245 (49%)	96 (32%)	2.04 (1.51, 2.75)		
**(e) Kerosene-cooking and/or lighting category**							
14	Kerosene-cooking or lighting (KCL)	No	218 (44%)	201 (67%)	1	5, 14, 15, 16	–
		Yes	282 (56%)	99 (33%)	2.62 (1.95, 3.54)		

*1^3^; 2^59^; 3^4^; 4^9^; 5^60^; 6^13^; 7^34^; 8^39^; 9^37^; 10^61^; 11^12^; 12^20^; 13^14^; 14^23^; 15^10^; 16^58^.

Specifically, AOR analysis (by considering all variables) was undertaken using a logistic model to show the association when variables coexist. This study has employed a data set of 800 respiratory patients. Finally, the study has applied the logistic model to identify the independent variables that significantly influence the incidences of TB in respiratory patients.

A strategy to regulate the significant variables can reduce the potential spread of TB. Most of the subjects (more than 75%) were from Kanpur and nearby districts (Kannoj, Kanpur, Kanpur Dehat, Lucknow and Unnao).

## Methodology

The first step was to obtain the approval for the questionnaire and protocol for conducting the survey from the ‘Institutional Ethics Committee (IEC) for Research Involving Human Subjects’ at the Indian Institute of Technology Kanpur (IITK). The subjects were informed about the purpose of the study, maintaining the confidentiality of data/identity, expected benefits of participation, and duration of study to ensure that participation in the study was entirely voluntary. The questionnaire was developed both in English and Hindi languages. Before conducting the survey, duly handwritten and self-attested consent was taken from all the subjects. There is no data/information in this article which reveals identity of subjects. The authors confirm that all experiments were performed in accordance with relevant guidelines and regulations of IEC (http://www.iitk.ac.in/dord/institutional-ethics-committee-iec).

All subjects were examined for sputum positive pulmonary tuberculosis by the two authors (Dr. Subodh Katiyar and Dr. Sandeep Katiyar), Chest and TB physicians. Then we had two streams of subjects, one who had the TB (referred to as Cases) and those who did not have the TB (referred to as Controls). The subjects from the two streams were surveyed face to face from November 2016 to March 2017. Of 800 total subjects, 500 (Cases) had TB and 300 (Control) did not have TB.

### Development of the questionnaire

A questionnaire (Supplementary Table S1) was developed to include the questions on independent variables, which may influence or associate with the prevalence of TB^21,38^. In the family category, there were five independent variables, namely, residence location (RUR; rural), family members in 1200 sq. ft. of area (FMW) (https://www.commonfloor.com/dda-mig-flats-delhi/povp-50k1kg), TB in the family (TBF), crowding per room (CPR), and smoking by a family member (SFM). In the personal category, there were four independent variables, gender (GEN), age (AGE), education (EDU) and smoking (SMO).

We defined the independent variable workplace (WPL) in the occupation category depending on where a person spent at least 8 h in a day. The question on WPL had multiple responses; schools, office, home, farms, construction and factory (mostly sugar mill, a cotton mill and glass cutting). The multiple responses were clubbed into two groups: clean environment workplace (CEW; office, farm and school)^52^ and polluted environment workplace (PEW; construction, factory, home (for housewives)).

In indoor air, exposures to fine particles are from the combustion of household fuels and poor ventilation in the kitchen^10,18^. Both kitchen ventilation and household combustions associate with TB, especially in women^14,15,18,53^. In this context, data were compiled under the category kitchen, which had three independent variables, namely, (i) kitchen location (KLO) (partitioned inside the house (PIH) or non-partitioned inside the house (NPIH)), (ii) cooking fuel uses (CFU) (LPG or solid fuels) and (iii) ventilation in the kitchen (VEN) (at least one window of size 2ft by 2ft or no window). The last independent variable was kerosene in cooking and/or lighting (KCL) (Table 1).

### Statistical analysis

#### UORs-based approach

The significance of UOR was examined based on z-test at the confidence interval (CI) of 95% i.e. significance level (P) of 0.05 (*p* ≤ 0.05), which measures if the odd ratio is statistically significant to show that exposure has indeed caused the outcome (i.e., TB in this study).

#### Logistic regression and strategy to regulate significant variables

UORs reported in many studies^12,13,15,23,34,35,37,54^ at best compare two variations in one independent variable. Realistically, all the variables are likely to coexist and should be considered in one go. Besides examining the UORs for each independent variable, we have used the logistic regression (also known as the *logit model*)^45^ to consider all independent variables as coexisting for their association with TB.

In the logistic regression, the dependent variable takes the binary form, generally in epidemiology and medical studies^4,55^ and independent variables can take on continuous and/categorical realizations^45,56^. The dependent variable *Y* has two possible values *y* = 1 (person having TB) and *y* = 0 (person not having TB). The transformed varaiable *TB (x)* is the natural log of the odds ratio of *y*_*i*_ = 1 versus *y*_*i*_ = 0 that is the log of odds of TB occuring relative to not occuring in the respiratory patients. There were 14 independent variables (*x*_*j*_; *j* = 1,…,14) (Table 1).

The logistic model was used to understand the relationship between the dependent variable and one or more independent variables by estimating probabilities using logistic regression to predict the likelihood of occurrence of an event (i.e., *y* = 1). The logistic regression uses the maximum likelihood technique to estimate the coefficients of independent variables. The rest of the analysis, significance and model acceptance tests are similar to the general linear regression.

We have used the logistic regression at three levels: (i) consider all 14 independent variables, (ii) develop a smaller model using step-wise regression (enter and remove the variable at 0.15 level of significance), and (iii) examine the model from (ii) to rebuild the model only for significant variables. The suitability of the model and significance of the variables were examined from changes in the natural log-likelihood (log (L)) levels. For the logistic regression, we have used SYSTAT (version 11) software.

The above modelling exercise will identify significant variables that will determine the probability of getting TB. Specifically, regulation or elimination of significant variables has been evaluated in the districts (most subjects came to CCC) in terms of reducing TB cases.

## Results and discussion

The subjects in this study consist of 500 TB cases (males 270 (54%) and females 230 (46%)) and 300 controlled cases (males 193 (64%) and females 107 (36%)). Among all TB patients, the TB stage was: 58% primary, 26% secondary and 16% tertiary.

Supplementary Table S2 presents descriptive statistics, the mean and standard deviation of variables. The mean value among all variables varies from 0.33 to 0.69. The highest mean value is for EDU (0.69) and the minimum for FMW (0.33). Supplementary Table S3 presents the correlation coefficient (listwise positive matching). The correlation among the variables was generally less than 0.5 and we did not expect the problem of collinearity in the variables.

### UORs analysis

An analysis of UORs with 95% confidence interval (CI) for independent variables and inferences of previous studies are given in Table 1.

#### Family category

The variation in RUR and SFM did not show any statistically significant difference in UORs. The significant variables those showed association with TB include: TBF (UOR 1.73 (1.27–2.35), FMW (UOR 1.55 (1.14–2.09)) and CPR (UOR 1.34 (1.00–1.78)). TBF appears to be an important variable as TB is a contagious disease and spreads through the air medium. The literature suggests that all eight studies show the TBF as a significant variable (Table 1).

Out of 12 previous studies in this category, number of studies showing the variable as significant and non-significant are (shown in parenthesis): RUR (3, 4), FMW (2, 0), TBF (8, 0), CPR (3, 6) and SFM (3, 3) (Table 1). It is seen from the literature (Table 1) that results of UORs of independent variables are not consistent among the studies, except for TBF.

#### Personal category

The variables GEN (UORs 1.53 (1.14, 2.06)), EDU (UORs 1.49 (1.08, 2.05), SMO (UORs 1.42 (1.05, 1.91) and AGE (UORs 1.36 (1.02, 1.81)) were significant (Table 1).

Although the percentage of female subjects was lower than the males, the females have a high risk of TB which could be due to the exposure to fumes of cooking from solid fuels which are analyzed later in the kitchen category. Interestingly, variable EDU was significant, suggesting illiteracy relates to TB. SMO appears to be an important variable as smoking alters the human immune response and causes multiple defects in immune cell^57^. The literature suggests that all nine studies show the variable as significant (Table 1).

Out of 13 previous studies in the personal category, the number of studies showing the variable as significant and non-significant are (both shown in parenthesis): GEN (3, 2), AGE (3, 2), EDU (3, 1) and SMO (9, 0) (Table 1). It is seen that except for SMO, the UORs for other variables are not consistent among the earlier studies (Table 1).

#### Occupation category

The variable WLP showed statistically significant UOR, suggesting that polluted work environment associates with TB. Two previous studies for this variable adjudged it significant (Table 1).

#### Kitchen category

The significant variables VEN, KLO and CFU are dominating with UORs 2.04 (1.51, 2.75), 1.66 (1.24, 2.21) and 1.62 (1.21, 2.17) respectively. It may be noted that all these variables relate to indoor air pollution, most significant being ventilation, impacting the women (housewives) who have shown a greater likelihood of getting TB. It is seen from the previous studies (Table 1) that results of UORs of independent variables are not consistent among the studies, except for CFU.

#### Use of kerosene-cooking and lighting category

The variable KCL shows a statistically significant UORs 2.62 (1.95, 3.54); this association is well–established in the literature^11,58^. Out of four previous studies in this category, all have shown this variable as significant (Table 1). It is seen from the previous studies that the results of UORs of independent variables are not consistent among the studies and their inferences differ (Table 1). The inconsistency among studies using UOR does not give confidence to policymakers as to which variable (s) should have priority for control.

### Logistic regression and interpretation

As a first step, we have considered all 14 independent variables (Table 1) in the full model in the logistic regression. The model with coefficients, standard errors (s.e.), wald statistics (i.e. coefficient/ its s.e.), a measure of log-likelihood log (L), *p*-value and AOR (with 95% CI) is given in Supplementary Table S4. It may be noted that (+) coefficients (or AOR > 1) show that the variable positively relates to the probability of getting the TB and (−) coefficients (or AOR < 1) suggest a negative association with the probability of getting TB. However, it is important to observe the significance of the independent variable.

**Figure 1:**
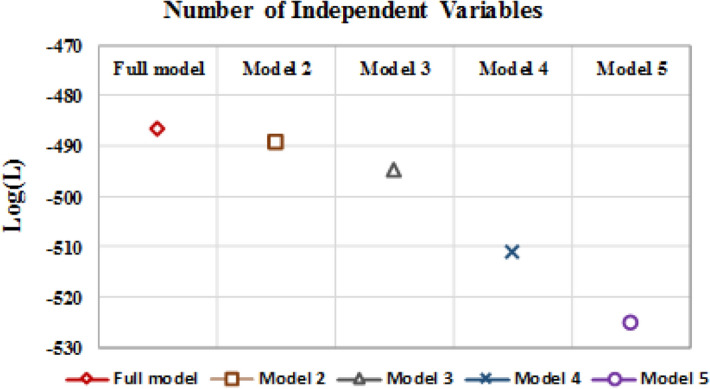
Log (L) Level for different models. (Models with variables: Model 1: Full Model (14 variables); Model 2: Stepwise (KCL, VEN, GEN, SMO, TBF, & WPL); Model 3: KCL, VEN, GEN and WPL; Model 4: VEN, GEN and WPL and Model 5: GEN and WPL.

The maximum log (L) was − 486.54 (Fig. 1) for the full model. It implies that as we drop any independent variable, the maximum log (L) will decrease. However, one has to see if the decrement in log (L) is marginal, then possibly the variable could be dropped, and we can obtain an equally good/acceptable model having fewer variables.

It may be noted that the independent variables (FMW, TBF, CPR, GEN, AGE, EDU, SMO, WPL, KLO, CFU, VEN and KCL) showed significant UOR (Table 1). However, when all variables were examined as group in the logistic model using AOR, some of them (RUR, FMW, CPR, SFM, AGE, EDU, WPL, KLO and CFU) lost their significance (Supplementary Table S4).

As the second step, we performed step-wise logistic regression to make the model smaller by dropping insignificant variables (as determined by stepwise regression). At the same time, we did not compromise the model predictively (i.e. Reduction in log (L)). The following model (Eq. (1)) was obtained, and in the process, eight independent variables got dropped. The estimated coefficients and their statistical significance are given in Supplementary Table S5.

1 $$TB\left( x \right) = - 1.450 + 0.848 \times KCL + 0.529 \times VEN + 0.541 \times GEN - 0.383 \times SMO - 0.349 \times TBF + 0.271 \times WPL$$

It is to be noted that for the smaller model (Eq. (1)), the log (L) slightly decreased (i.e. 0.53%) to − 489.114. If we now examine UORs in Table 1, the important looking variables (FMW, CPR, AGE, EDU, KLO and CFU) have been dropped in the model obtained from the stepwise logistic regression, without compromising on log (L).

In the next level of analysis, we see that SMO has a negative coefficient (Eq. (1)). It implies that smoking may reduce the probability of getting TB, which is not correct, therefore, even if this variable is significant (*p*-value = 0.026), we should drop it. This unusual situation may have occurred as the subjects may not have told the truth because of the stigma it carries about smoking or at the time of the survey they might have left smoking.

In light of the above, we have further dropped two variables, SMO, and TBF which had negative coefficients. However, in the process, the log (L) marginally decreased to − 494.83 (a decrease of 1.16%) (Fig. 1). The new model (refereed as subset model) (Eq. (2)) that is obtained now is smaller in number of independent variables (i.e. four). The other statistical details of variable significance are given in Supplementary Table S6.

2 $$TB\left( x \right) = - 2.691 + 0.875 \times KCL + 0.560 \times VEN + 0.451 \times GEN + 0.321 \times WPL$$

In the above model, all the independent variables are significant and positively relate to the incidences of TB. To further examine if any of the significant independent variables can be further dropped and how would the log (L) will decrease, the variable KCL was dropped on purpose.

It is to be noted that by dropping KCL, the log (L) sharply decreased to − 510.98 (3.16%) (Fig. 1). To further elucidate the importance of variable, if we also drop VEN, the log (L) decreases to − 525.11 (2.22%) (Fig. 1). These decrements signify that we should not drop any variable from the Eq. (2).

### Model performance: full model vs subset model

**Figure 2:**
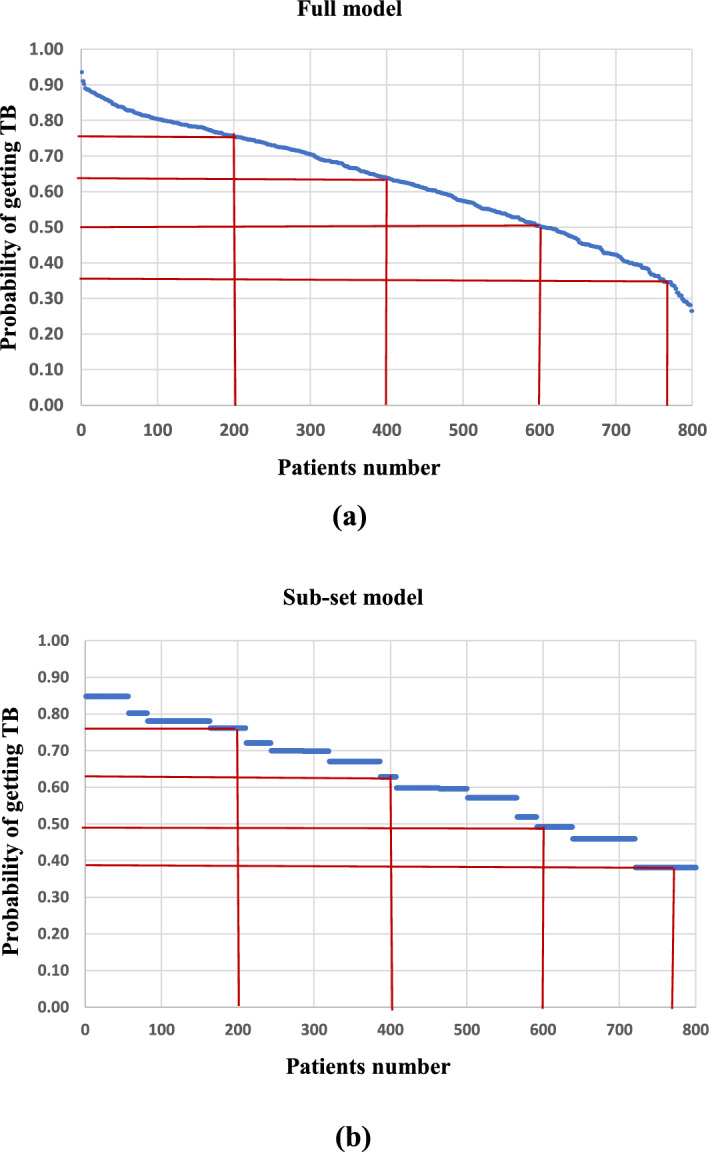
Probability plot for (**a**) full model, and (**b**) sub-set model (Eq. (2)).

Before developing any strategy based on the subset model, its suitability is examined against the full model. The probability of each subject having the TB (i.e., *y*_*i*_ = 1) was estimated. For a better comparison, the estimated probabilities are plotted in descending order for both the models (Fig. 2).

The maximum probability for a subject getting TB was 0.94 for the full model and 0.85 for the subset model. The estimates of probability for the percentile of number of subjects are nearly the same for both the models (Fig. 2, Supplementary Table S7). To further establish the subset model, a simulation was undertaken by generating the 5000 binary random numbers for 14 independent variables and estimating the probabilities of getting TB for 5000 subjects as per the full model (14 variables) and the subset model (Eq. (2)). It is seen for 5000 simulated subjects, the estimated probabilities from the full and subset models are comparable (Supplementary Fig. S1, Supplementary Table S8). It is concluded from the discussion on log (L) (Fig. 1) and probability estimates that the subset model with a fewer variable performs as good as the full model and can be used for developing strategies for control of TB.

### TB control strategy

#### Kerosene uses and TB incidences

If kerosene uses can be controlled it is likely to have a significant impact on reducing incidences of TB. It also makes sense from the point of administrative control and management as kerosene supply is well-structured and households using kerosene are well-documented. In India, kerosene is widely used for cooking (4.57 million tons/year) and in lamps for lighting (1.45 million tons/year)^24^; kerosene burns at least 4–5 h a day in lamps. There is a large emission of PM_2.5_ and black carbon (BC) from kerosene burning^24,62–64^ Out of 901 Gg/year BC emission in India, about 110 Gg/year is from kerosene burning^65^.

Several studies suggest that emissions from kerosene lamps are the precursor for TB infection and disease^22,66,67^. According to the WHO^68^, exposure to BC can lead to cardiopulmonary morbidity and mortality. WHO also suggests that BC may act as a universal carrier of chemicals of varying toxicity to the lungs^68^. Fullerton et al. have shown a larger loading of particulates in alveolar macrophages in the persons using kerosene lamps than those using candles or electric lamps^22,69^. Other emissions from kerosene combustion include carbon monoxide, nitrogen dioxide, carbon dioxide, sulfur dioxide, formaldehyde, and various VOCs (volatile organic carbons)^64^. Out of all sources (vehicles, industry, and other fuels), the emission factor for BC from kerosene lamps is nearly two orders of magnitude higher at 84 gm/kg of kerosene^65^. Literature^22,62,70^ also suggests that kerosene has other air pollution issues and requires replacement, especially in developing countries.

**Figure 3:**
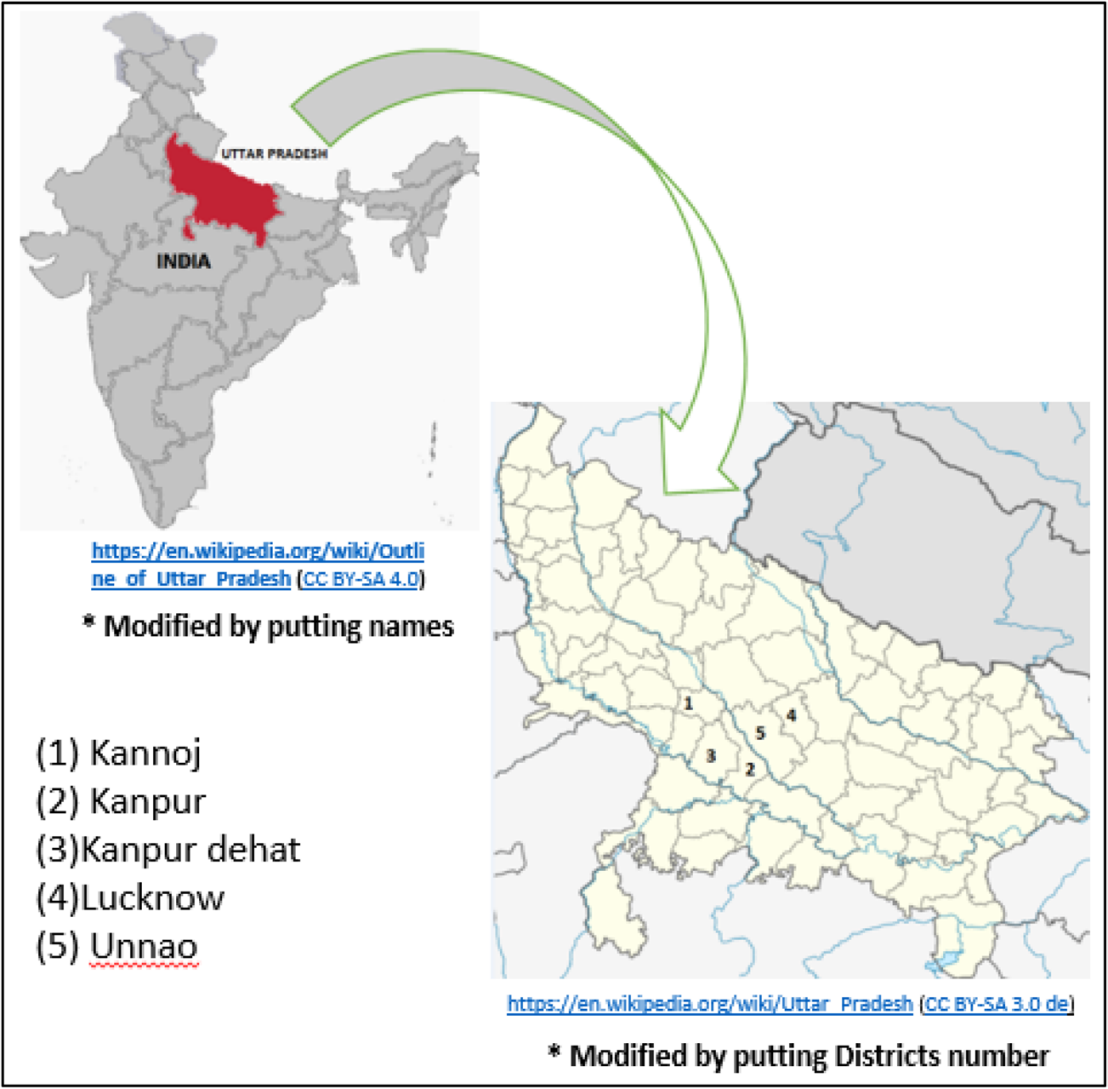
Districts from where most patients visited CCC.

**Figure 4:**
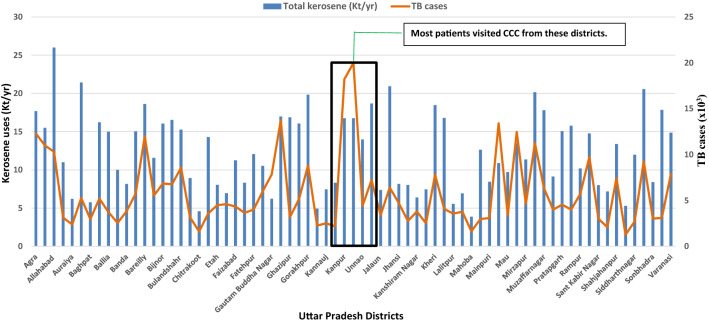
District-wise Uses of kerosene^65^ (Kt/year) and TB cases in the state of Uttar Pradesh^51^.

It was decided to examine the strategy for replacing 50% kerosene from the districts of Kannoj, Kanpur, Kanpur Dehat, Lucknow and Unnao in the State of Uttar Pradesh (Fig. 3), which had the majority of subjects visiting the CCC. The district-wise consumption of kerosene in kilo-tons/year (Kt/year) and incidences of TB in all 71 districts, including five highlighted districts, is shown in Fig. 4. The correlation coefficient between kerosene uses and TB incidences was 0.88 for five districts and 0.57 for all districts.

#### Reduction of kerosene uses on TB incidences

As a strategy to prevent the incidences of TB, we have developed a scenario that 50% of kerosene uses is reduced for cooking and lighting purposes. As a first step, we randomized data set off 800 subjects to avoid any biases of selecting the 50% cases of kerosene uses, which would be replaced with other clean fuel. Using the subset model (i.e. Eq.  (2)), the mean probability of getting TB for those subjects who now use only 50% of kerosene is reduced by 11.57%. The estimated reduction in probability of TB incidences using the full model was 11.39%. To establish the contextual reduction in TB incidences in larger population, a simulation was undertaken by generating 5000 binary random numbers for 14 independent variables and estimating the reduction in mean probabilities of getting TB (in the subjects using kerosene). The estimated reduction was 12.20% for the full model and 13.29% for the subset model (Supplementary Fig. S1). The reduction in the probabilities for the simulated subjects is consistent for full and subset model and compares well with the reduced probability derived for 800 subjects, based on recorded data.

The study has estimated the reduction in probability of TB incidences in respiratory patients if 50% kerosene uses is stopped. Arguably the findings can convince the policy makers to accelerate replacing kerosene from households with the clean fuel like liquid petroleum gas (LPG) or petroleum natural gas (PNG) and rural electrification. In the Indian context, the *Ujjwala* scheme of LPG supply and *Saubghagya* scheme of rural electrification must be strengthened and expanded.

While this study evidenced the potential prevention of TB incidences, the other benefits include better indoor and outdoor air quality and increased immunity for other diseases and reduction in mortality. Findings of this study could possibly apply to other south Asian countries which are similar in culture, urban-poor divide, economic conditions and high prevalence of TB. The policymakers can take science-based decisions for major interventions for control of TB. Therefore, we focused the study on helping policymakers understand the causal linkage responsible for TB and encourage them for long-term planning and investment.

## Conclusion

The current research has looked at the association between TB incidences and 14 independent variables by analyzing the unadjusted odds ratio (UOR) and adjusted odds ratio (AOR) in 800 respiratory patients. The variables include: urban/rural, persons in 1200 sqft area, TB in family, crowding, smoking (family member), gender, age, education, smoking, workplace, kitchen location, cooking fuel, ventilation, and kerosene uses. It was concluded that inferences from UORs were not consistent among the studies and thus, actions taken may not be effective in all situations. It was concluded that variables must be dealt with as a group and their significance should be based on the adjusted odds ratio (AOR). For example, independent variables TB in family, kitchen location, and occupation showed significant UORs but lost their significance when examined through AOR, derived from the logistic model. In the logistic model, only four variables kerosene uses, kitchen ventilation, gender and workplace, were found significant and adequate compared to a full model of 14 variables.

It was concluded that uses of kerosene (AOR 2.62 (1.95, 3.54)) and emissions thereof consistently related to the incidences of TB. The linkage between incidences of TB and kerosene uses was quantified and it was concluded that a 50% reduction in kerosene uses could prevent 13.29% of existing TB cases.

In this study, most subjects (more than 75%) were from Kanpur and nearby districts (Kannoj, Kanpur, Kanpur Dehat, Lucknow and Unnao) in Uttar Pradesh, India. The findings of this study, in a strict sense, may not hold true for the rest of the districts in Uttar Pradesh or other states in India.

The conclusion of kerosene and TB linkage can be useful to other south Asian countries similar in culture, urban-poor divide, economic conditions and high prevalence of TB. The science-based findings can prompt the policymakers to control variables responsible for TB and inspire them for long-term planning and investment.

## Supplementary Information

Supplementary Information.
