# Supplementary material for: Logistic regression analysis of environmental and other variables and incidences of tuberculosis in respiratory patients
Source: Sci Rep. 2020 Dec 14;10:21843. doi: 10.1038/s41598-020-79023-5 (PMC7736574; doi:10.1038/s41598-020-79023-5)

Logistic Regression Analysis of Environmental and other Variables and Incidences of Tuberculosis in Respiratory Patients

Ashutosh K Pathak[1], Mukesh Sharma[1*], Subodh K Katiyar[2], Sandeep Katiyar[2] and Pavan K Nagar[1]

[1]Department of Civil Engineering and Centre for Environmental Science and Engineering, Indian Institute of Technology Kanpur, Kanpur, India-208016

[2]Dr. S K Katiyar Chest Care Centre, Kanpur, India-208016

*Corresponding Author: Mukesh Sharma, Department of Civil Engineering, Indian Institute of Technology Kanpur, Kanpur, India-208016, Email: mukesh@iitk.ac.in

Supplementary Information

Submitted to

Scientific Reports

Table S1: Condensed questionnaire

(a) Family Category

S No.	Independent variable (abbreviations)	Groups within variable
**1**	Residence – Urban/Rural **(RUR)**	Urban
		Rural
**2**	Family members in 1200 sq. ft. of area **(FMW)**	≤5
		>5
**3**	TB in family **(TBF)**	No
		Yes
**4**	Crowding per room **(CPR)**	≥ 2
		> 2
**5**	Smoking by family member **(SFM)**	No
		Yes

(b) Personal Category

S No.	Independent variable (abbreviations)	Groups within factors
**6**	Gender **(GEN)**	Male
		Female
**7**	AGE	≤30
		>30
**8**	Education **(EDU)**	Literate
		Illiterate
**9**	Smoking **(SMO)**	No
		Yes

(c) Occupation Category

S No.	Independent variable (abbreviations)	Groups within factors
**10**	Workplace **(WPL)**	CEW
		PEW

(d) Kitchen Category

S No.	Independent variable (abbreviations)	Groups within factors
**11**	Kitchen Location **(KLO)**	PIH
		NPIH
**12**	Cooking Fuel usage **(CFU)**	LPG
		Solid fuels
**13**	Ventilation **(VEN)**	Yes
		No

(e) Use of Kerosene

S No.	Independent variable (abbreviations)	Groups within factors
**14**	Kerosene-Cooking and/or Lighting **(KCL)**	No
		Yes

Table S2: Descriptive statistics, mean and standard deviation of independent variables

	**TB**	**RUR**	**FMW**	**TBF**	**CPR**	**SFM**	**GEN**	**AGE**	**EDU**	**SMO**	**WPL**	**KLO**	**VEN**	**CFU**	**KCL**
**Mean**	0.63	0.53	0.33	0.36	0.46	0.44	0.58	0.43	0.69	0.37	0.38	0.58	0.57	0.38	0.52
**Standard Dev**	0.48	0.50	0.47	0.48	0.50	0.50	0.49	0.50	0.46	0.48	0.49	0.49	0.50	0.49	0.50

Table S3: Correlation coefficient (listwise positive matching) matrix of varaibales

	Y	RUR	FMW	TBF	CPR	SFM	GEN	AGE	EDU	SMO	WPL	KLO	VEN	CFU	KCL
Y	0.63														
**RUR**	0.33	0.53													
**FMW**	0.18	0.20	0.33												
**TBF**	0.25	0.19	0.09	0.36											
**CPR**	0.27	0.26	0.21	0.14	0.46										
**SFM**	0.28	0.21	0.12	0.17	0.21	0.44									
**GEN**	0.34	0.30	0.19	0.20	0.29	0.24	0.58								
**AGE**	0.25	0.24	0.16	0.14	0.21	0.18	0.21	0.43							
**EDU**	0.41	0.39	0.25	0.26	0.34	0.29	0.41	0.35	0.69						
**SMO**	0.25	0.19	0.10	0.17	0.16	0.18	0.28	0.12	0.25	0.37					
**WPL**	0.21	0.21	0.16	0.12	0.19	0.15	0.22	0.21	0.35	0.12	0.38				
**KLO**	0.39	0.29	0.18	0.23	0.25	0.27	0.33	0.23	0.37	0.24	0.18	0.58			
**VEN**	0.32	0.33	0.20	0.19	0.29	0.24	0.33	0.26	0.45	0.20	0.25	0.27	0.57		
**CFU**	0.21	0.24	0.18	0.10	0.21	0.12	0.23	0.20	0.30	0.11	0.19	0.15	0.28	0.38	
**KCL**	0.27	0.30	0.23	0.17	0.28	0.20	0.31	0.25	0.38	0.19	0.22	0.26	0.34	0.29	0.52

Table S4: Full model: Coefficients and significance

Categorical values encountered during processing are:
TBNONTB (2 levels)
0, 1
Binary LOGIT Analysis.
Dependent variable: TBNONTB
Input records: 800
Records for analysis: 800
Sample split
Category choices
0 (REFERENCE) 300
1 (RESPONSE) 500
Total : 800
Log Likelihood: -486.540

S No.	Variable	Coefficient	Standard error	Wald	*p*-value	95.0 % bounds		
						AOR	Upper	Lower
0	Constant	-1.522	0.880	-1.730	0.084			
1	RUR	-0.081	0.159	-0.510	0.610	0.922	1.259	0.676
2	FMW	0.135	0.175	0.772	0.440	1.145	1.613	0.812
3	TBF	-0.367	0.171	-2.149	0.032	0.693	0.968	0.496
4	CPR	-0.036	0.163	-0.219	0.827	0.965	1.327	0.702
5	SFM	0.167	0.161	1.037	0.300	1.182	1.622	0.862
6	GEN	0.579	0.170	3.404	0.001	1.785	2.492	1.279
7	AGE	0.171	0.165	1.037	0.300	1.186	1.639	0.859
8	EDU	0.076	0.193	0.392	0.695	1.079	1.575	0.739
9	SMO	-0.380	0.175	-2.167	0.030	0.684	0.964	0.485
10	WPL	0.212	0.175	1.211	0.226	1.237	1.744	0.877
11	KLO	-0.172	0.170	-1.007	0.314	0.842	1.176	0.603
12	VEN	0.521	0.170	3.060	0.002	1.684	2.352	1.206
13	CFU	-0.210	0.190	-1.105	0.269	0.811	1.176	0.559
14	KCL	0.884	0.174	5.093	0.000	2.420	3.401	1.722

Table S5: Stepwise regression: Coefficients and significance

Categorical values encountered during processing are:
TBNONTB (2 levels)
0, 1
Binary LOGIT Analysis.
Dependent variable: TBNONTB
Input records: 800
Records for analysis: 800
Sample split

Category choices
0 (REFERENCE) 300
1 (RESPONSE) 500
Total : 800

Log Likelihood: -489.114

S No.	Variable	Coefficient	Standard error	Wald	*p*-value	95.0 % bounds		
						AOR	Upper	Lower
0	Constant	-1.450	0.575	-2.522	0.012			
1	KCL	0.848	0.158	5.379	0.000	2.335	3.180	1.714

2	VEN	0.529	0.160	3.301	0.001	1.698	2.324	1.240
3	GEN	0.541	0.165	3.276	0.001	1.718	2.374	1.243
4	SMO	-0.383	0.172	-2.228	0.026	0.682	0.955	0.487
5	TBF	-0.349	0.166	-2.098	0.036	0.706	0.977	0.509
6	WPL	0.271	0.159	1.705	0.088	1.311	1.789	0.960

Table S6: Subset model: Coefficients and significance

Categorical values encountered during processing are:
TBNONTB (2 levels)
0, 1
Binary LOGIT Analysis.
Dependent variable: TBNONTB
Input records: 800
Records for analysis: 800
Sample split
Category choices
0 (REFERENCE) 300
1 (RESPONSE) 500
Total : 800
Log Likelihood: -494.830

S No.	Variable	Coefficient	Standard error	Wald	*p*-value	95.0 % bounds		
						AOR	Upper	Lower
0	Constant	-2.691	0.441	-6.104	0.000			
1	KCL	0.875	0.156	5.607	0.000	2.399	3.257	1.767
2	VEN	0.560	0.159	3.523	0.000	1.750	2.390	1.282
3	GEN	0.451	0.156	2.889	0.004	1.570	2.131	1.156
4	WPL	0.321	0.157	2.049	0.040	1.378	1.874	1.014

Table S7: Perentile probability distribution of getting TB from full and subset model (800 subjects)

**Percentile of number of subjects**	**Probability of getting TB (Full model)**	**Probability of getting TB (Subset model))**
25 %	0.77	0.76
50 %	0.66	0.63
75 %	0.50	0.49
95 %	0.36	0.38

Table S8: Perentile probability distribution of getting TB from full and subset model (5000 simulated subjects)

**Percentile of number of subjects**	**Probability of getting TB (Full model)**	**Probability of getting TB (Subset model))**
25 %	0.77	0.72
50 %	0.67	0.63
75 %	0.56	0.52
95 %	0.41	0.38

Figure S1: Probability distribution of getting TB in 5000 simulated subjects: full versus subset model

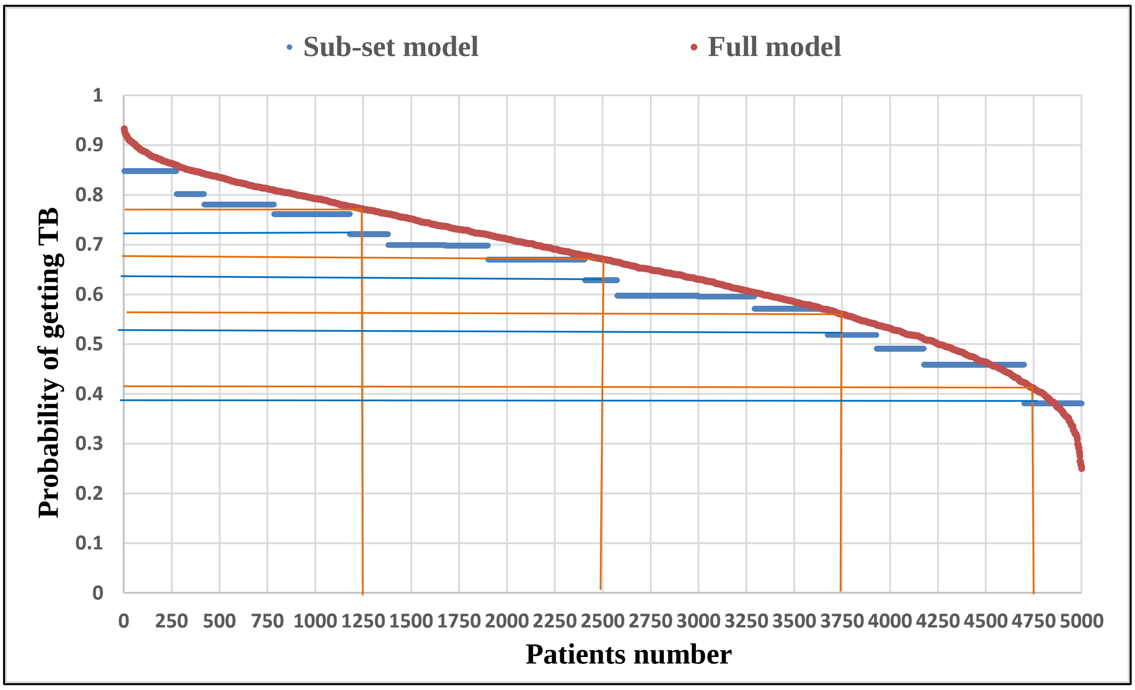

Supplement: Supplementary file 1 — Supplementary Information. [file 41598_2020_79023_MOESM1_ESM.pdf]
